# Sample-efficient parameter exploration of the powder film drying process using experiment-based Bayesian optimization

**DOI:** 10.1038/s41598-022-05784-w

**Published:** 2022-02-08

**Authors:** Kohei Nagai, Takayuki Osa, Gen Inoue, Takuya Tsujiguchi, Takuto Araki, Yoshiyuki Kuroda, Morio Tomizawa, Keisuke Nagato

**Affiliations:** 1 Department of Mechanical Engineering, The University of Tokyo, Bunkyo-ku, Tokyo 113-8656 Japan; 2 Department of Human Intelligence Systems, Kyushu Institute of Technology, Fukuoka, 808-0135 Japan; 3 Department of Chemical Engineering, Kyushu University, Fukuoka, 819-0395 Japan; 4 Faculty of Mechanical Engineering, Kanazawa University, Kanazawa, Ishikawa 920-1192 Japan; 5 Department of Systems Integration, Yokohama National University, Yokohama, Kanagawa 240-8501 Japan; 6 Department of Materials Science and Chemical Engineering, Yokohama National University, Yokohama, Kanagawa 240-8501 Japan

**Keywords:** Design, synthesis and processing, Fuel cells, Characterization and analytical techniques, Computational methods

## Abstract

Parameter optimization is a long-standing challenge in various production processes. Particularly, powder film forming processes entail multiscale and multiphysical phenomena, each of which is usually controlled by a combination of several parameters. Therefore, it is difficult to optimize the parameters either by numerical-model-based analysis or by “brute force” experiment-based exploration. In this study, we focus on a Bayesian optimization method that has led to breakthroughs in materials informatics. Specifically, we apply this method to exploration of production-process-parameter for the powder film forming process. To this end, a slurry containing a powder, polymer, and solvent was dropped, the drying temperature and time were controlled as parameters to be explored, and the uniformity of the fabricated film was evaluated. Using this experiment-based Bayesian optimization system, we searched for the optimal parameters among 32,768 (8^5^) parameter sets to minimize defects. This optimization converged at 40 experiments, which is a substantially smaller number than that observed in brute-force exploration and traditional design-of-experiments methods. Furthermore, we inferred the mechanism corresponding to the unknown drying conditions discovered in the parameter exploration that resulted in uniform film formation. This demonstrates that a data-driven approach leads to high-throughput exploration and the discovery of novel parameters, which inspire further research.

## Introduction

### Background

The examination of production processes is necessary for attaining the inherent characteristics of any novel material and for realizing the desired performance of products. Among such processes, powder film forming is a fundamental process that must be used in either prototyping or mass production to fabricate functional devices such as rechargeable batteries^1^, fuel cells^2–4^, solar cells^5^, and water electrolysis systems^6^.

The powder film forming process is a production process that is used to manufacture thin-film products from powdery materials, such as carbon or ceramic powders. It consists of several major subprocesses (typically dispersion, mixing, coating, and drying), all of which have a significant impact on the performance of the final product. However, similar to most production processes, the powder film forming process is controlled by a number of parameters, and the phenomena entailed in the process are complex. Efforts to elucidate the details are still ongoing^7–9^. Given the complexity of the phenomena, it is almost impossible to build an adequate model that describes the entire process^10^, which indicates that numerical approaches are impractical. In addition, the large number of parameters hinder the evaluation of all combinations of parameters using a brute-force approach. Determining the parameters of the process depends on empirical rules and the extensive effort and skill of engineers and researchers; therefore, it cannot be guaranteed that any adopted parameter set would be optimal. Therefore, a non-parametric, sample-efficient parameter exploration method should be developed for the powder film forming process.

### Related work

A similar problem has persisted in the field of materials science. However, in recent years, the application of machine learning to materials exploration has achieved some success^11–14^, enabling high-throughput exploration of, particularly, alloy compositions^15^, which was previously based on empirical or brute-force methods. In particular, in previous studies using active learning, including Bayesian optimization (BO), efficient searches were performed with a small number of samples (a few percent of the entire candidates)^16–18^. BO is a powerful optimization method that often uses Gaussian process regression (GPR) to predict the relationship between parameters and performance^19,20^. GPR can estimate a model considering uncertainty, and BO using GPR is effective for the efficient exploration of a large parameter space^17^. The application of BO has produced remarkable results in a wide range of fields. For example, in the fields of robotics, BO has been applied to learn a controller for a bipedal robot^21^ and robotics grasping^22,23^. Prior work also demonstrated that BO is effective for transfer learning in natural language processing^24^. More recently, BO was applied to optimize the hyperparameters of deep learning for AI, which beat a professional human Go player^25^. Therefore, BO is also expected to be a suitable approach for production processes, in which it is challenging to search for the optimal process parameters manually and heuristically. In fact, BO has been applied to search for parameters in processes such as polymer fiber synthesis^26^, TiO_2_ film depositions^27^, and gas atomization of alloy powders^28^. However, there were some challenges that needed to be addressed, e.g., the explored parameter space was not large enough for practical use and the product evaluation was subjective and not sufficiently reliable. When applying BO to a production process that does not have any suitable numerical model, it is necessary to repeat the experiments (fabrication and evaluation of the test specimens) during the optimization. Because the exploration in BO progresses based only on the results of the experiments conducted without assuming any prior knowledge, the experiments used to train the optimization system must be reproducible and quantitative. Therefore, most attempts to apply BO methods to materials development have used numerical simulations instead of experiments^29–32^. Nevertheless, pioneering research using industrial robots to overcome the problem of reproducibility and to conduct searches autonomously are being conducted for catalytic^33^, organic^34^, and inorganic materials^27^.

### Aim of this study

Film manufacturing processes that utilize raw materials in powder form are often used in the fabrication of polymer electrolyte fuel cell (PEFC) electrodes^35–37^. A PEFC is a type of fuel cell that has attracted considerable attention as a power source for automobiles and for portable use because of its low operating temperature. The catalyst layer in PEFC electrodes consists of carbon particles with Pt loaded on the surface and a polymer. The polymer acts as an ionomer that provides a conduction pathway for protons during power generation; Nafion™ is generally used as the polymer. In the dispersion step, these materials are dispersed in water and alcohol. Carbon particles that are several tens of nanometers in diameter are widely used, and the particles agglomerate in the solvent to form secondary particles that are several hundreds of nanometers in diameter^38^. The ionomers are generally well dispersed in alcohol; however, similar to carbon particles, the ionomers agglomerate and become stable in a colloidal state. The ionomers also agglomerate around the secondary particles of carbon to form shells^39,40^. The degree of dispersion and agglomeration of the materials depend on the fabrication steps used. This ultimately affects the microstructure and distribution of components after drying. Notably, these features affect the power generation performance of PEFCs depending on electrochemical reaction activity, gas diffusivity and proton conductivity; therefore, improvements to the fabrication process of electrodes are essential for increasing the efficiency of PEFCs^36,41,42^. The dispersion and agglomeration of material particles also cause surface defects like cracks on the fabricated film^43^. It is possible to evaluate the power generation performance indirectly by evaluating the easily observable defects like cracks^44^. Therefore, in this study, cracks in the film are selected as targets for optimization. In the industry, PEFC catalyst layers are fabricated by brush coating, doctor blade, inkjet printing, or other methods^45,46^; however, we conducted experiments using drop casting, which can easily produce multiple samples with a small amount of slurry. Prototyping with a small amount of material is also meet the demands of the product development stage. Drop casting also suffers from the aforementioned problems of film forming and is appropriate for use in a case study as a simple and fundamental example of film forming^47^.

**Figure 1:**
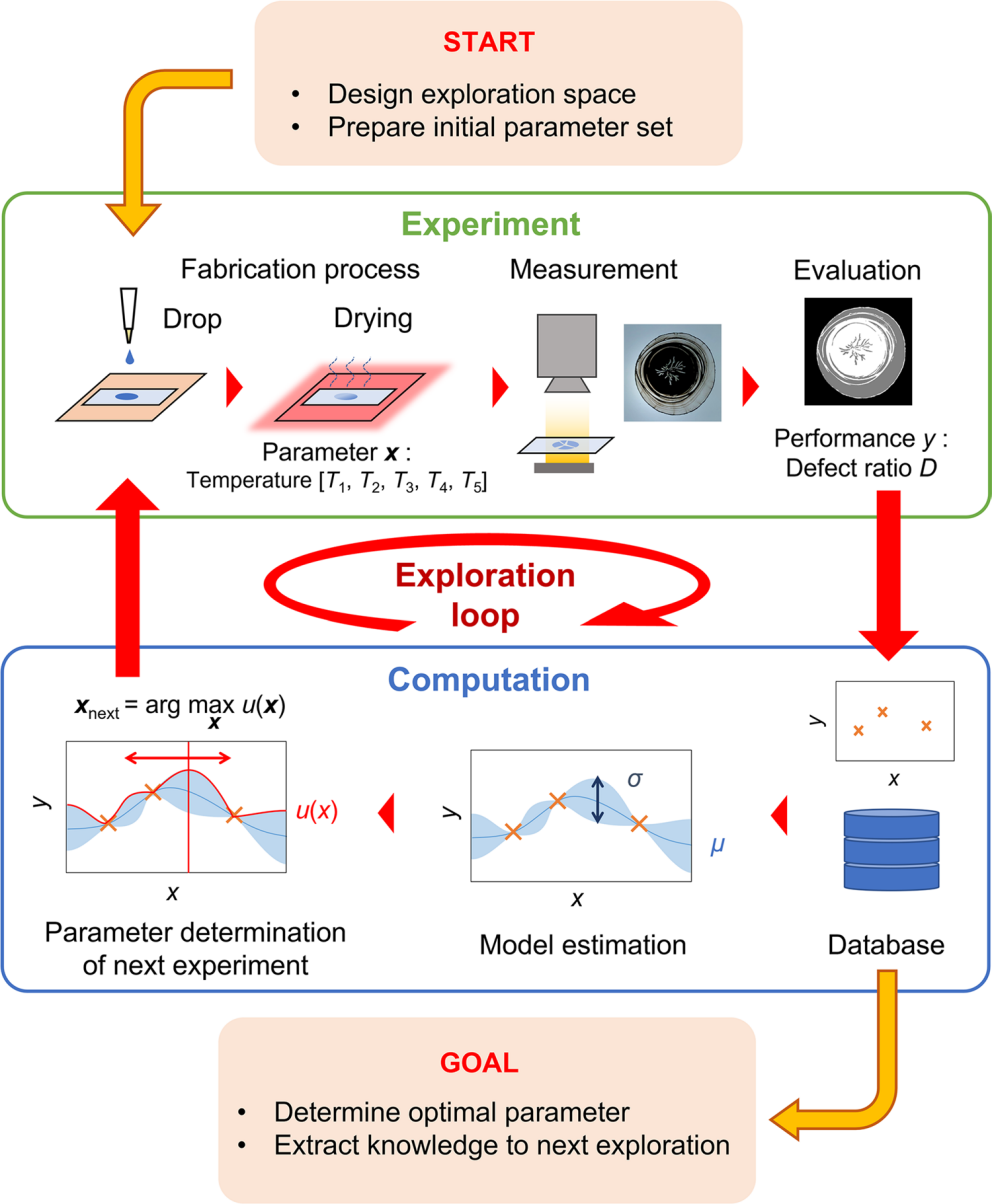
Overview of our process parameter exploration system using BO. First, the experiments were conducted under random parameters to collect data. The model was estimated with uncertainty, performance was predicted, and parameters for the next experiment were determined. The experiments were conducted using the proposed parameters, and we added the observed performance to the database. This loop was repeated to progress the exploration.

In this study, we demonstrate the optimization of the drying process in PEFC electrode fabrication. Figure 1 shows a conceptual diagram of the proposed experiment-based BO. The material slurry was dried while controlling the heating temperature, and the homogeneity of the resulting electrode film was quantitatively evaluated. Based on the obtained datasets of temperature profiles and electrode film states, the machine learning system estimated a surrogate model and proposed the parameters under which more homogeneous films were expected to be formed. Then, experiments were conducted accordingly. By repeating the experiment cycles, we could explore the parameters with a smaller number of experiments than that required in conventional methods. Moreover, the detailed mechanism of the drying phenomenon could be determined. In our previous study^48^, we confirmed that the parameters determined by BO were partially applicable for this process. However, the effectiveness of the parameter exploration system was not evaluated. In this study, we further discuss the process phenomena using the exploration results and evaluate the performance of the parameter exploration system to demonstrate its effectiveness using BO. This approach is useful for improving the efficiency of parameter exploration of production processes, and it is the first step towards elucidating the complicated phenomena entailed in such processes.

## Experimental procedure

To estimate the surrogate model for the first iteration of the search, we conducted 30 experiments using random temperature profiles (see “Methods” section for details). Although a difference in the initial states has some effect on the number of experiments required for convergence^26^, the random profiles were adopted to evaluate the exploration from an initial state without bias. Thirty microliters of the slurry were dropped onto a glass slide placed on a heater and dried while controlling the temperature of the heater. The temperature profile had five time steps, and each time step was assigned one of the eight temperature levels. There were 8^5^ = 32,768 candidate combinations in the exploration space. In the initial dataset preparation phase, experiments were conducted with 30 temperature profiles that were randomly selected from all the possible combinations. For each fabricated electrode, cracks and exceedingly thin regions were extracted based on the brightness values of the surface images, and the proportion of the detected areas to the total area where the dropped ink spread was evaluated as the “defect ratio”.

Using the obtained initial data as a starting point, we conducted parameter exploration via BO. To predict the defect ratio, we built a surrogate model using GPR^49^, which is commonly used in BO^17,29^. After each fabrication, an acquisition function was calculated for each temperature profile using the predicted values of the surrogate model, and the temperature profile with the highest score was adopted as the subsequent experimental parameter set. The acquisition function was set to the upper confidence bound (UCB)^50–52^. The UCB strategy balances exploration and exploitation during optimization. In BO, the superiority of the UCB strategy over other strategies, such as expected improvement and probability of improvement has been reported^21,53^. Although the advantages of other acquisition functions have been suggested^54^, investigating the best acquisition function is out of the scope of this study. We performed the fabrication process ten times to evaluate the defect ratio, and the experimental parameter set for this procedure was selected each time.

## Results

### Observation of the fabricated electrodes

**Figure 2:**
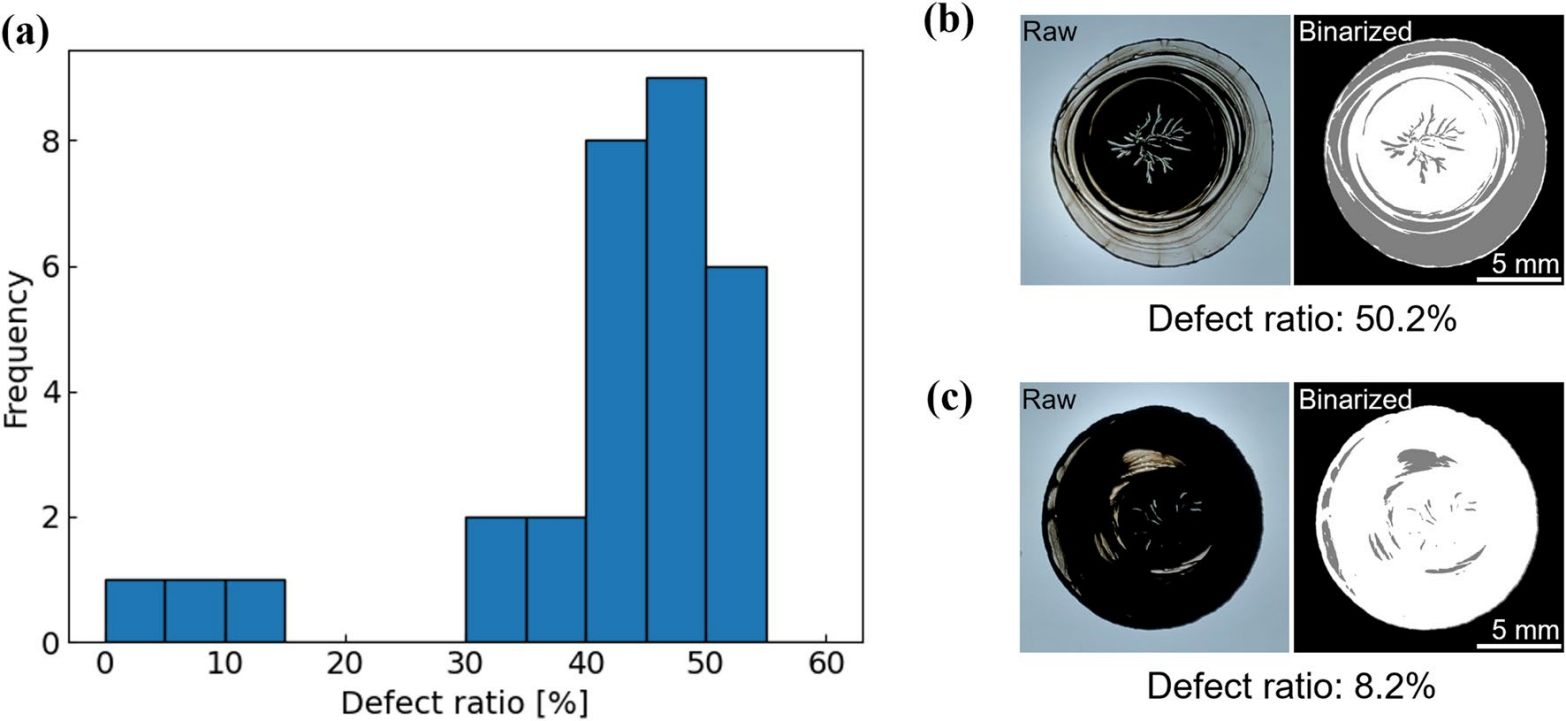
Results of the experiments under random parameters. (**a**) Distribution of the observed defect ratios and typical appearances of samples with (**b**) high defect ratio and (**c**) low defect ratio. In (**b**) and (**c**), the raw images are shown on the left side, and the images with the defect areas detected by binarization are shown on the right side (adapted with permission from ref. ^48^ Copyright (2021) the Electrochemical Society).

For most of the temperature profiles, defect ratios of 30% to 50% were observed; however, in rare samples (3 out of 30 samples), the defect ratios were below 15% (Fig. 2a). In the samples with high defect ratios, clear radial cracks at the center of the electrodes and an exceedingly thin region at the periphery were typically observed, and carbon was unevenly deposited in concentric circles (Fig. 2b). By contrast, the samples with a low defect ratio had no thin regions at the periphery, and sparse cracks were observed; however, they were not clearly radial (Fig. 2c). The diameter of the fabricated films was approximately 13 mm and the thickness was approximately 30 μm at the thickest area.

### Parameter exploration

**Figure 3:**
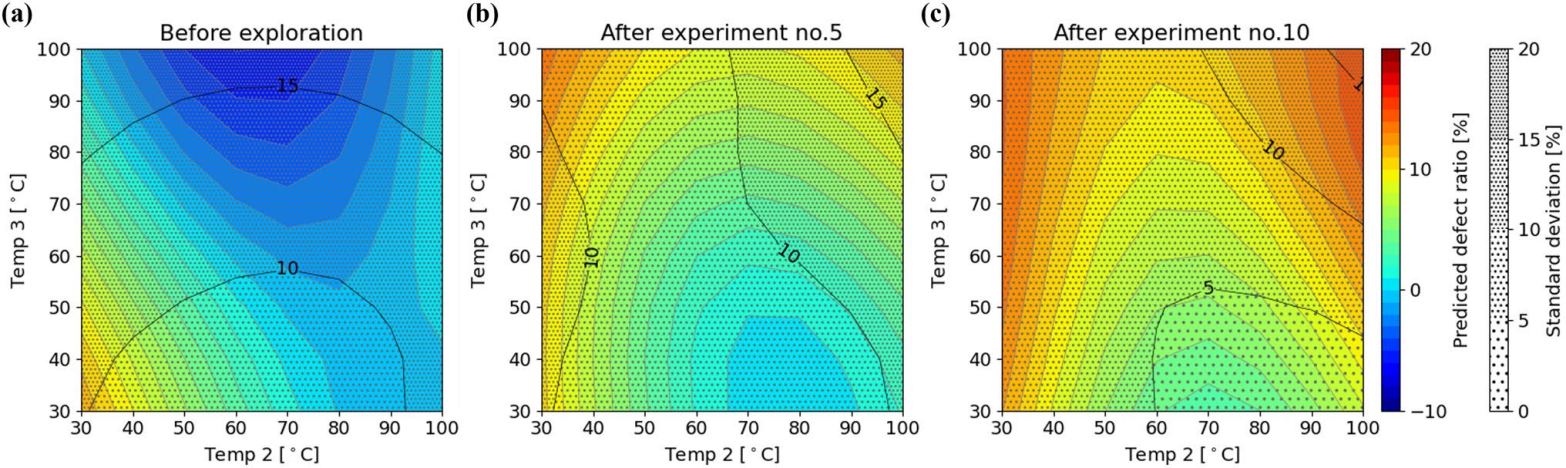
Prediction maps of the relationship between the temperature profiles during drying and defect ratios (**a**) before exploration, (**b**) after five experiments, and (**c**) after ten experiments of exploration. To visualize this, the second and third temperature profiles have been extracted and shown. Moreover, the uncertainty *σ* is shown by hatching. Since the prediction range of the defect ratio was not restricted, negative defect ratios are seen in (**a**), which can be interpreted as corresponding to parameter spaces where lower (closer to 0%) defect ratios are more likely to be obtained.

**Figure 4:**
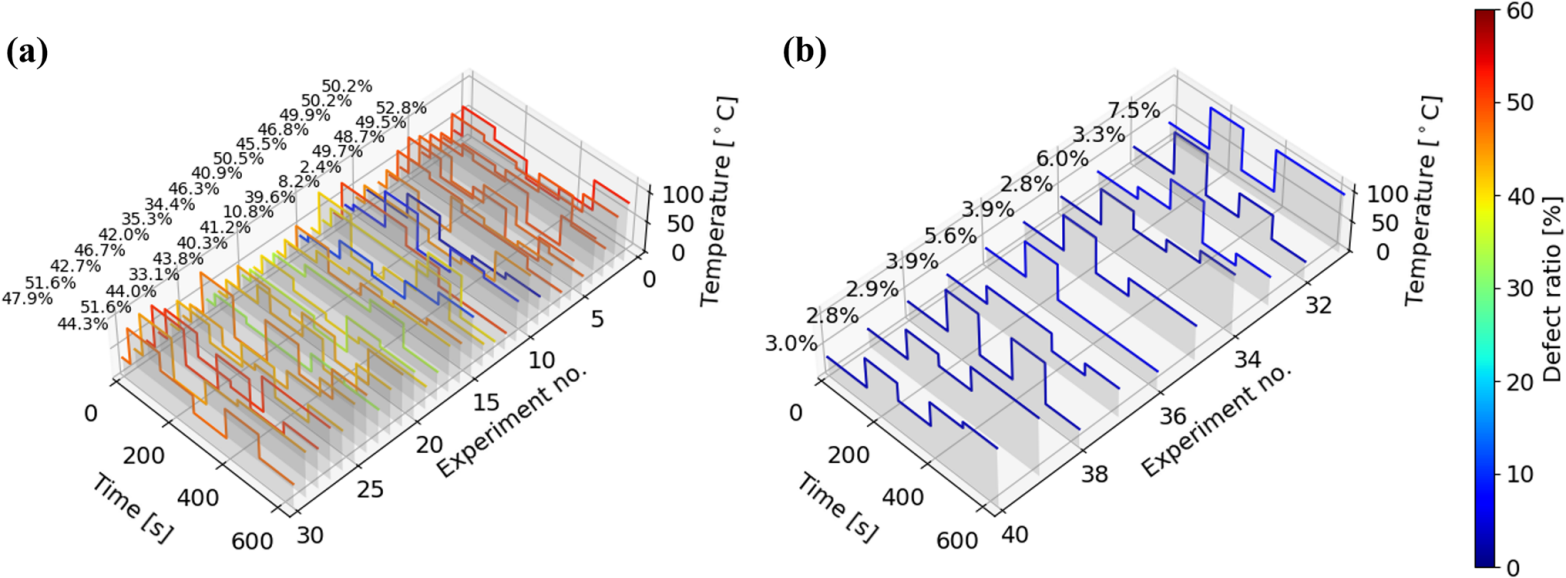
Temperature profiles and defect ratios for each experiment in the (**a**) initial data preparation and (**b**) exploration steps.

**Figure 5:**
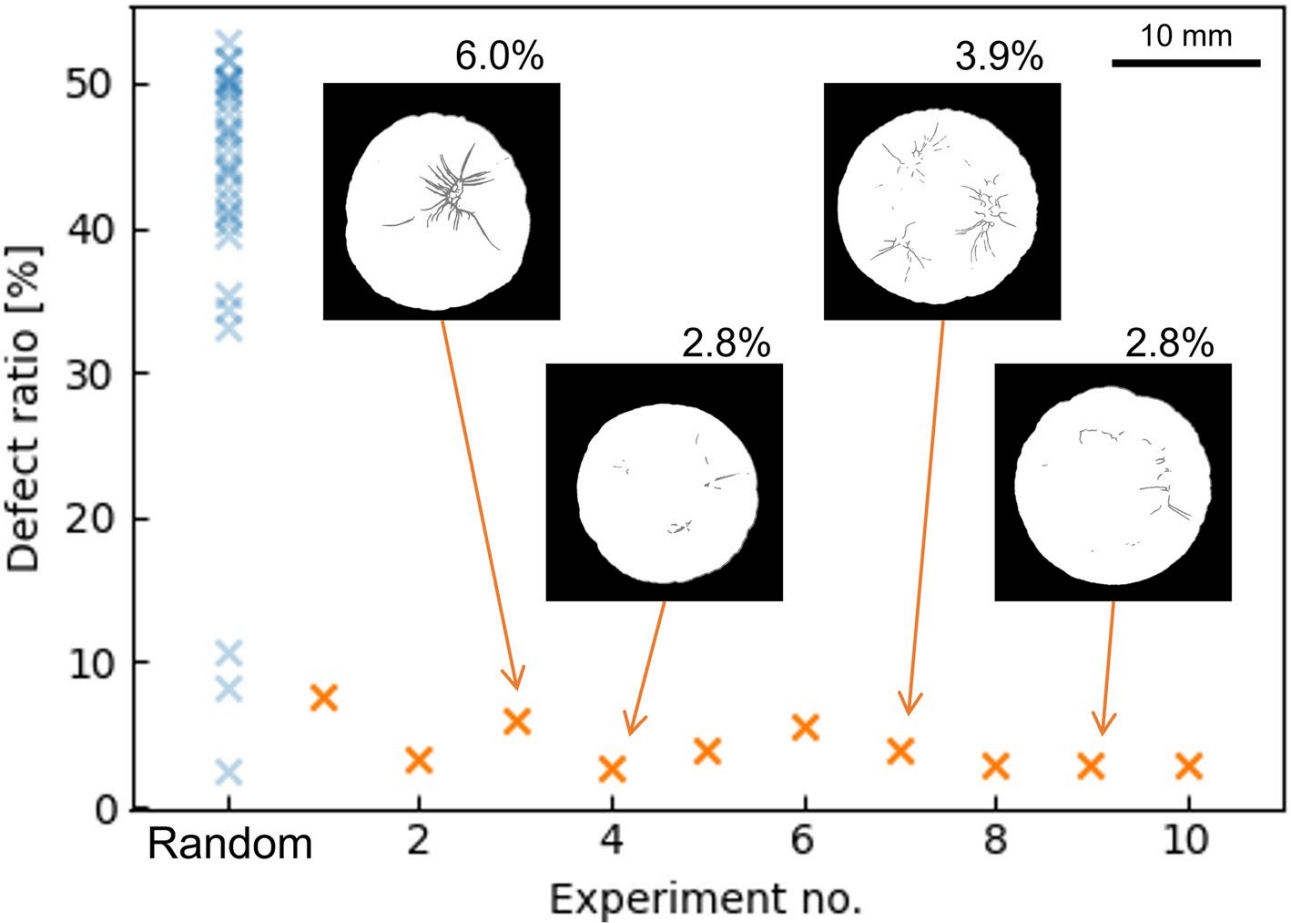
Comparison of the defect ratios observed in the exploration with the defect ratios in the initial data. The latter are shown in blue on the left side of the plot. The typical binarized appearances of the samples obtained in the exploration are shown in the plot.

Figure 3 shows the relationship between the temperature profiles and the defect ratios predicted by GPR during the exploration, using two of the five parameters for visualization. From the figures, it can be confirmed that as the search progresses, the regions wherein low defect ratios are predicted become more limited, while the variance of the predictions become smaller. The temperature profiles and observed defect ratios in each experiment are shown in Fig. 4, and the comparison of the defect ratio distribution with the initial data is shown in Fig. 5. In the exploration phase, the defect ratios were less than 10% for all the tested parameter sets, and the lowest value was 2.8%, which was obtained in experiments four and nine. Moreover, a lower defect ratio was observed as the exploration progressed. These results suggest that the application of BO makes parameter exploration more efficient than iterations of random experiments. During the initial data preparation phase, wherein the process parameters are selected randomly, even if a temperature parameter that achieves a relatively low defect ratio is found, it is challenging to guarantee the optimality of the temperature profile because of the large search space. However, exploration using the combination of GPR and UCB strategy found the temperature profile that outperforms other temperature profiles, and the convergence of the defect ratio can be a criterion for stopping the exploration in practice. Performance prediction maps obtained during the exploration, such as the one shown in Fig. 3, provide a basis for the relative usefulness of the proposed process parameters. Moreover, the maps are clues that lead to the elucidation of the phenomena occurring in the process of interest, as discussed in the subsequent section.

### Examination of the drying mechanism based on knowledge extracted from the regression results

**Figure 6:**
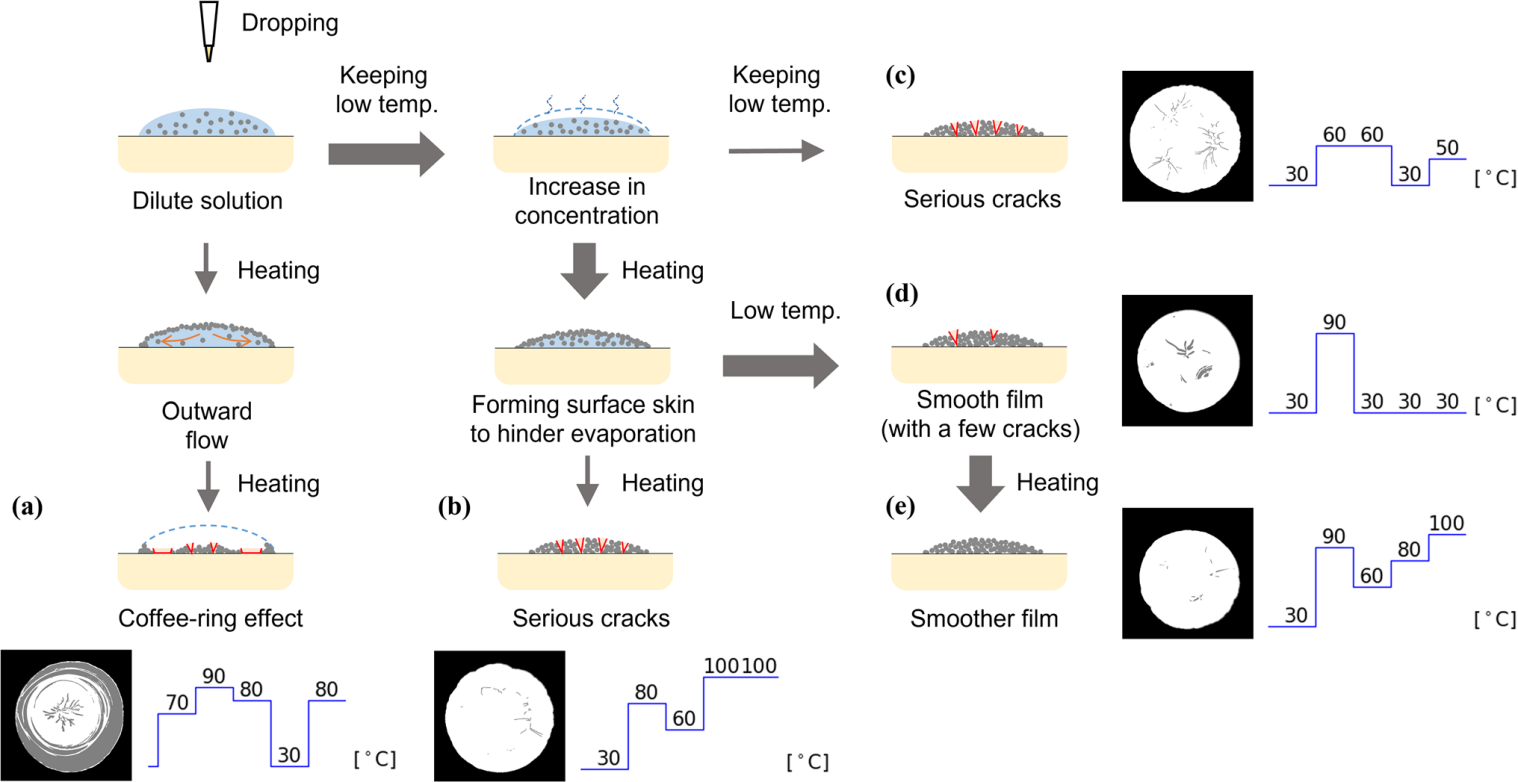
Illustration of the hypothesis of the mechanism of how a low–high–low temperature profile reduces defects during drying. The temperature profile discovered may correspond to a very limited process window.

Upon examining the relationship between the temperature profiles and defect ratios, presented in Fig. 4, low defect ratios tended to be observed in the temperature profiles wherein the temperature of the first step was low (30 °C), that of the second step was relatively high (above 60 °C), and that of the third step was also relatively low (below 60 °C). This trend is also confirmed by the performance prediction map obtained after ten experiments (Fig. 3c). Comparing experiments five and six, it seems that raising the temperature after lowering it once has the effect of reducing the defect ratio. In industrial powder drying processes, it is common to employ a single drying temperature, rate of temperature increase, and holding time, without finely controlling the temperature profile. Therefore, it is likely that the low–high–low (–high) temperature profile proposed and validated based on the machine learning system cannot be discovered via trials following conventional methods. The phenomena occurring in the drying process are discussed as follows (Fig. 6).

Immediately after the drop, the slurry had a low solid phase ratio, and maintaining a low temperature caused the solvent to evaporate gently, leading to an increase in the solid phase ratio. If the slurry was heated to a high temperature immediately after the drop, the carbon particles would have flowed outward, forming an uneven distribution, and the final film would have had an uneven thickness. In addition, concentric and uneven precipitation of carbon particles is observed in the samples heated rapidly immediately after dropping (Fig. 6a), and this non-uniformity is commonly observed upon the drying of dilute solutions^55–57^. Once the solid phase ratio is sufficiently increased by drying at a low temperature, accelerated evaporation by increasing the temperature would not cause uneven precipitation. Nonetheless, it was possible for the evaporation of the solvent to progress on the surface of the liquid film, forming a skin of carbon particles bonded to each other and suppressing intensive evaporation. During the drying of colloidal solutions, the packing of particles on the surface generally lowers the evaporation rate^58^. A low evaporation rate may be realized if a low temperature is maintained throughout the drying process; nevertheless, the evaporation rate can be lower if the aforenoted skin is formed by heating once. Moreover, the defect ratio was not substantially low under the continuous-low-temperature profiles (Fig. 6c). After the skin is formed, the temperature is decreased, and the remaining solvent is gently evaporated from the inside of the film, followed by complete drying of the film. This results in a crack-free film with a uniform thickness (Fig. 6d). The effect of heating on the formation of the skin and the subsequent defect reduction was confirmed in our previous study^48^. In the samples that continued to be heated at high temperatures even after the skin was formed, radial cracks were observed. This can be attributed to the rapid evaporation of the solvent, inevitably leading to the fixing of the position of the carbon particles and causing residual stress in the film (Fig. 6b). Heating at the last stage of drying reduces the defect ratio, likely because precipitated Nafion™ can deform near the glass-transition temperature and relax the stress (Fig. 6e)^59^. Considering the drying mechanism discussed above, the temperature profile, which enabled low defect ratios, discovered by the machine learning system was in an exceedingly limited process window and was not expected to be found as an extension of the traditional drying methods.

## Discussion

We demonstrated that appropriate process windows can be discovered with a high throughput and a small number of samples by repeating experiments using the parameters provided by the machine learning system. The total number of experiments conducted in this study was 40, corresponding to approximately 0.1% of all the possible parameter combinations. Therefore, the exploration was 1,000 times more efficient than evaluating all the parameters individually. Even if a researcher or an engineer, rather than a machine learning system, was to consider previous results and propose subsequent experimental parameters for each experiment, it would be almost impossible for a human to understand the distribution of the predicted performance in a five-dimensional (in this study) parameter space. Thus, such an efficient exploration would not be possible. Furthermore, while conventional parameter adjustments in trial-and-error methods require experts to analyze the results for each experiment, in our method, human intervention is required only for setting the search space and for the analysis of the entire exploration result. Thus, in addition to the time cost, the human cost can be significantly reduced. The advantage of machine learning systems becomes even more significant when the number and/or range of parameters is expanded. However, this does not undermine the value of specialists who have a deeper understanding and keen insight into the processes and phenomena involved, and their focus remains downstream. The map of the relationship between process parameters and performances, obtained via exploration using BO, contains a large amount of useful information. By examining this map closely, specific inferences regarding the phenomena that govern a process can be made, as we have done in this study. Such analyses are expected to lead to subsequent higher-throughput explorations. This study is a pioneering example of a “human-in-the-loop” system, wherein artificial intelligence sensitizes human intelligence to repeat high-throughput explorations.

Another highlight of this study is the application of existing powerful machine learning methods to process exploration by setting up appropriate optimization parameters and experimental/evaluation techniques for the target process. Although simultaneous exploration of numerous parameters is acceptable, parameters that do not affect the process or are difficult to control may increase the cost of the experiments. This contradicts the original purpose of a highly efficient search and makes the interpretation of the proposed parameter sets and the resulting maps difficult. In addition, if the evaluation criterion is not set appropriately, the experiments become more difficult and the measurement error may crucially affect the prediction results. We address these limitations by devising experimental equipment and using image processing, which are also areas that require the knowledge of specialists.

The experiment-based process parameter exploration demonstrated in this study based on PEFC electrode film-forming is a pioneering example of the “process informatics” methodology that follows materials informatics. Process informatics methods can be developed in various directions, such as by expanding the number and/or range of parameters, applying such methods to slightly different conditions, and applying such methods to processes in other fields. In this study, we used five-dimensional parameters and a single evaluation criterion. However, considering the demonstrated exploration efficiency, it is acceptable to explore larger parameter spaces and adopt more complex evaluation criteria. Moreover, even if the process preconditions that are not used for exploration change slightly, the trends of the performance maps are presumed to have a commonality. Therefore, more efficient exploration can be performed by referring to the results of previous explorations in similar processes^60^. The application of process informatics is not limited to powder film forming, and this method can be applied to other industrial processes to obtain outstanding results. This study is only a case study of a specific process for a certain material, and is insufficient to examine the robustness of the method for other materials and processes or the influence of the properties of the input data (such as number, error, and density of the data). Nevertheless, process informatics is useful as a method for the rapid optimization of processes associated with novel materials proposed in materials informatics. Once a sufficiently high-throughput process informatics method is established, the bottleneck in materials development may become the prototyping of the materials.

## Methods

### Preparation of slurry for dropping and fabrication of samples

The specimen slurry comprised carbon particles (Vulkan® XC-72R, Cabot) and 5% Nafion™ dispersion solution (DE520 CS type, FUJIFILM Wako Chemical Corporation). These materials were added to water and 2-propanol at an ionomer/carbon weight ratio of 0.8, solid phase ratio of 0.1, and water ratio of 0.3. In this study, we did not conduct a power generation evaluation; therefore, carbon particles without Pt catalyst were used. Just before the experiments, this slurry was diluted three times with IPA and was subsequently subjected to 10 min of stirring using a planetary centrifugal mixer (mixing at 2,000 rpm for 5 min and defoaming at 2,200 rpm for 5 min) and 10 min sonication at 45 kHz.

The slurry was dropped onto a glass slide on a heater using a motorized pipette (dPette 30–300, DLAB) handled by a robot arm (DOBOT Magician, Shenzhen Yuejiang Technology). We used an automated dropping system for reproducible dropping. In each experiment, 30 μL of the slurry was dropped and dried. The heater temperature was maintained at 30 °C even before the drop, and one of eight temperature levels, with equal intervals from 30 to 100 °C, was applied every 120 s for 600 s in the range of 30–630 s after the drop. After 630 s of drying following the drop, the fabricated electrode was removed from the heater and observed using transmitted light. Regarding the quantitative evaluation, sample images were processed using OpenGL. The films were photographed at 504 × 504 pixels using transmitted light. The captured area corresponded to approximately 16 mm^2^. The defect ratio was calculated by separating the area where the dropped ink spread (droplet area) from the background and the detection of the defect areas in the droplet area. First, the raw images were binarized, and the contour of the droplet was detected according to the brightness values. To prevent cracks reaching the outer edge of the droplet from being treated as background, the contour was smoothed by expanding the contour line width once and then shrinking the width. The interior area of the resulting contour was evaluated as the droplet area. The black regions in Fig. 2b and c were excluded as background by the above process. The droplet areas were binarized again, and the areas with high brightness values, that is, areas where the formed electrode layer was thin or almost absent and most of the reference light was transmitted, were detected as defects, as demonstrated by the gray regions in Fig. 2b and c. All samples were captured and analyzed under the same conditions, and the binarization thresholds were set at 150 out of 256 shades for background separation and 50 out of 256 shades for defect detection. The proportion of the defect area to the droplet area was defined as the defect ratio.

### Gaussian process

We used GPR to estimate the relationship between the temperature profiles and defect ratios. The GPR algorithm used in this study is available in scikit-learn^61^.To estimate the relationship, we have to find the model $p(y|{\varvec{x}},{ \mathcal{D}})$, given a dataset ${\mathcal{D}} = \left\{ {{\varvec{x}}_{i} , y_{i} } \right\}_{i = 1}^{n}$, where ${\varvec{x}}$ is the parameter of the temperature profile and $y$ is the resulting defect ratio. Because the fabricated samples are observed with noise, the regression problem to solve is given by

1 $$\begin{array}{*{20}c} {y\left( {\varvec{x}} \right) = f\left( {\varvec{x}} \right) + \eta ,\quad \eta \sim \mathcal{N}\left( {0,\beta } \right),} \\ \end{array}$$

where we assume a zero mean Gaussian noise. By using GPR:

2 $$\begin{array}{*{20}c} {f\left( {\varvec{x}} \right) \sim \mathcal{G}\mathcal{P}\left( {\mu \left( {\varvec{x}} \right),k\left( {{\varvec{x}},\user2{x^{\prime}}} \right)} \right),} \\ \end{array}$$

where $\mu ({\varvec{x}})$ is the mean function and $k({\varvec{x}}, {\varvec{x}}\boldsymbol{^{\prime}})$ is the covariance function of the Gaussian process. In this study, the covariance is modeled by the radial basis function (RBF):

3 $$\begin{array}{*{20}c} {k\left( {\varvec{x}, \user2{x^{\prime}}} \right) = C^{2} \exp\left( { - \frac{{\left| {\left| {{\varvec{x}} - \user2{x^{\prime}}} \right|} \right|^{2} }}{{2l^{2} }}} \right),} \\ \end{array}$$

where $C$ is a constant and $l$ is the length-scale parameter. Considering the initial conditions, we set $\mu \left( {\varvec{x}} \right) = 0$ as the mean function. The set of hyper parameters ${\varvec{\theta}} = \left\{ {C, l, \beta } \right\}$ can be optimized by maximizing the marginal likelihood given by

4 $$\begin{array}{*{20}c} {\log p\left( {{\varvec{y}}{|}{\varvec{\theta}}} \right) = - \frac{1}{2}{\varvec{y}}^{{\mathbf{T}}} \left( {{\varvec{K}} + \sigma^{2} {\varvec{I}}} \right)^{ - 1} \varvec{y} - \frac{1}{2}\log\left| {\left( {{\varvec{K}} + \sigma^{2} {\varvec{I}}} \right)} \right| - \frac{n}{2}\log\left( {2\pi } \right).} \\ \end{array}$$

In scikit-learn, the optimization of ${\varvec{\theta}}$ is implemented using the L-BFGS-B algorithm^62^ and executed for each regression. Given the dataset ${\mathcal{D}}$, the covariance matrix between the previously observed ${\varvec{x}}$ and ${\varvec{y}}$ is given as

5 $$\begin{array}{*{20}c} {\varvec{K} = \left[ {\begin{array}{*{20}c} {k\left( {{\varvec{x}}_{1} ,{\varvec{x}}_{1} } \right)} & \cdots & {k\left( {{\varvec{x}}_{1} ,{\varvec{x}}_{n} } \right)} \\ \vdots & \ddots & \vdots \\ {k\left( {{\varvec{x}}_{n} ,{\varvec{x}}_{1} } \right)} & \cdots & {k\left( {{\varvec{x}}_{n} ,{\varvec{x}}_{n} } \right)} \\ \end{array} } \right] + \beta \varvec{I}.} \\ \end{array}$$

The joint Gaussian probability of the dataset ${\mathcal{D}}$ and the prediction of $y^{ + }$, which is expected to be observed with a new parameter ${\varvec{x}}^{ + }$, is, assuming a zero mean prior, given by

6 $$\begin{array}{*{20}c} {\left[ {\begin{array}{*{20}c} {{\varvec{y}}_{1:n} } \\ {y^{ + } } \\ \end{array} } \right] \sim \mathcal{N}\left( {0,\left[ {\begin{array}{*{20}c} {\varvec{K}} & {\hat{\user2{k}}} \\ {\overline{\user2{k}}} & {k\left( {{\varvec{x}}^{ + } ,{\varvec{x}}^{ + } } \right)} \\ \end{array} } \right]} \right),} \\ \end{array}$$

with

7 $$\begin{array}{*{20}c} {\hat{\user2{k}} = \left[ {k\left( {{\varvec{x}}_{1} ,{\varvec{x}}^{ + } } \right) \cdots k\left( {{\varvec{x}}_{n} ,{\varvec{x}}^{ + } } \right)} \right]^{{\text{T}}} ,} \\ \end{array}$$

8 $$\begin{array}{*{20}c} {\overline{\user2{k}} = \left[ {k\left( {{\varvec{x}}^{ + } ,{\varvec{x}}_{1} } \right) \cdots k\left( {{\varvec{x}}^{ + } ,{\varvec{x}}_{n} } \right)} \right].} \\ \end{array}$$

Then, conditioning the Gaussian process on ${\varvec{x}}^{ + }$, the predictive posterior $p\left( {y^{ + } {|}{\varvec{x}},{\mathcal{D}}} \right)$ of ${\varvec{x}}^{ + }$ is given by the Gaussian:

9 $$\begin{array}{*{20}c} {p\left( {y^{ + } {|}{\varvec{x}},{\mathcal{D}}} \right) \sim \mathcal{N}\left( {\mu \left( {{\varvec{x}}^{ + } } \right),\sigma^{2} \left( {{\varvec{x}}^{ + } } \right)} \right),} \\ \end{array}$$

with the mean and variance calculated by

10 $$\begin{array}{*{20}c} {\mu \left( {{\varvec{x}}^{ + } } \right) = {\varvec{k}}^{{\text{T}}} {\varvec{K}}^{ - 1} {\varvec{y}}_{1:n} ,} \\ \end{array}$$

11 $$\begin{array}{*{20}c} {\sigma^{2} \left( {{\varvec{x}}^{ + } } \right) = k\left( {{\varvec{x}}^{ + } ,{\varvec{x}}^{ + } } \right) - {\varvec{k}}^{{\text{T}}} {\varvec{K}}^{ - 1} \varvec{k}.} \\ \end{array}$$

The obtained means $\mu \left( {{\varvec{x}}^{ + } } \right)$ and variance $\sigma \left( {{\varvec{x}}^{ + } } \right)$ were used to calculate the acquisition function.

### Acquisition function

In this study, since the objective is to minimize the defect ratio, the objective function $R\left( {\varvec{x}} \right)$ is given as follows:

12 $$\begin{array}{*{20}c} {R\left( {\varvec{x}} \right) = - D\left( {\varvec{x}} \right),} \\ \end{array}$$

where $D\left( {\varvec{x}} \right)$, which is an unknown function, is the value of the defect ratio in the experiment with parameter ${\varvec{x}}$. The UCB strategy was used to determine the temperature profiles of the experiments. The acquisition function $U\left( {\varvec{x}} \right)$ for the UCB strategy is given by

13 $$\begin{array}{*{20}c} {U\left( \varvec{x} \right) = \mu \left( {R\left( \varvec{x} \right)} \right) + c\sigma \left( {R\left( \varvec{x} \right)} \right),} \\ \end{array}$$

where $\mu \left( {R\left( {\varvec{x}} \right)} \right)$ is the mean value and $\sigma \left( {R\left( {\varvec{x}} \right)} \right)$ is the standard deviation of $R\left( {\varvec{x}} \right)$. The coefficient $c$ of $\sigma \left( {R\left( {\varvec{x}} \right)} \right)$ is a constant that determines the balance between exploration and exploitation. The UCB strategy shows good performance for various tasks when $c$ is in the range of 0.1 to 1^54^. In this study, we set $c = 0.5$.

## Data Availability

The data that support the findings of this study are available from the corresponding author on request.
